# Analysis of the Respiratory Activity in the Antarctic Yeast *Rhodotorula mucilaginosa* M94C9 Reveals the Presence of Respiratory Supercomplexes and Alternative Elements

**DOI:** 10.3390/microorganisms12101931

**Published:** 2024-09-24

**Authors:** Daniel Reyes-Rosario, Juan Pablo Pardo, Guadalupe Guerra-Sánchez, Héctor Vázquez-Meza, Georgina López-Hernández, Genaro Matus-Ortega, James González, Marcelo Baeza, Lucero Romero-Aguilar

**Affiliations:** 1 Departamento de Microbiología, Instituto Politécnico Nacional, Escuela Nacional de Ciencias Biológicas, Plan de Carpio y Plan de Ayala S/N Santo Tomás, Miguel Hidalgo, Mexico City C.P. 11340, Mexico; 2 Departamento de Bioquímica, Facultad de Medicina, Universidad Nacional Autónoma de México, Circuito Interior, Ciudad Universitaria, Coyoacán, Mexico City C.P. 04510, Mexico; 3 Departamento de Biología Celular, Facultad de Ciencias, Universidad Nacional Autónoma de México, Avenida Universidad 3000, Ciudad Universitaria, Coyoacán, Mexico City C.P. 04510, Mexico; james@ciencias.unam.mx; 4 Departamento de Ciencias Ecológicas, Facultad de Ciencias, Universidad de Chile, Santiago 7800003, Chile

**Keywords:** extremotolerant, NADPH dehydrogenase, OxPhos, mitochondrial respiratory chain

## Abstract

The respiratory activities of mitochondrial complexes I, II, and IV were analyzed in permeabilized *Rhodotorula mucilaginosa* cells and isolated mitochondria, and the kinetic parameters K_0.5_ and V_max_ were obtained. No difference in substrate affinities were found between mitochondria and permeabilized cells. The activities of the components of the mitochondrial respiratory chain of the Antarctic yeast *R. mucilaginosa M94C9* were identified by in-gel activity and SDS-PAGE. The mitochondria exhibited activity for the classical components of the electron transport chain (Complexes I, II, III, and IV), and supercomplexes were formed by a combination of the respiratory complexes I, III, and IV. Unfortunately, the activities of the monomeric and dimeric forms of the F1F0-ATP synthase were not revealed by the in-gel assay, but the two forms of the ATP synthase were visualized in the SDS-PAGE. Furthermore, two alternative pathways for the oxidation of cytosolic NADH were identified: the alternative NADH dehydrogenase and the glycerol-3-phosphate dehydrogenase. In addition, an NADPH dehydrogenase and a lactate cytochrome b2 dehydrogenase were found. The residual respiratory activity following cyanide addition suggests the presence of an alternative oxidase in cells.

## 1. Introduction

*Rhodotorula* species, classified within the Basidiomycota phylum, have been studied and isolated from diverse environments, including soil, ocean water, milk, food, and medical devices such as central venous catheters [1,2]. *Rhodotorula* is considered an extremotolerant organism that grows under cold climates, high osmolarity, and low nutrient availability, and even tolerates high concentrations of heavy metals such as Hg^2+^, Pb^2+^, and Cu^2+^ [3]. *Rhodotorula* species exhibit significant biotechnological potential. They produce pigments with applications in pharmaceuticals, food, animal feed, and aquaculture. Notably, certain pigments have demonstrated immune-stimulating properties against pathogens like bacteria or viruses [1]. Despite their advantageous features, *Rhodotorula* species, particularly *R. mucilaginosa, R. minuta,* and *R. glutinis,* have garnered attention as opportunistic pathogens, causing infections in immunocompromised individuals. Strains isolated from *R. mucilaginosa* infections have shown high resistance to antifungal agents like micafungin and fluconazole [4]. Because of its resistance to antifungal therapy, its oligotrophic characteristic, and its biotechnological impact, *R. mucilaginosa* has received extensive attention. However, little is known about their central metabolism. The expression of the genes related to glycolysis, the tricarboxylic acid cycle (TCA), and the oxidative phosphorylation (OxPhos), which belong to the central pathways of aerobic microorganism, has been studied in *R. mucilaginosa* [3]. Recently, the specific components of its electron transfer chain were elucidated, although the author did not report the presence of supercomplexes [5]. Thus, further understanding of the energy production mechanisms of *R. mucilaginosa* could have significant implications in various fields. 

This study focused on *R. mucilaginosa* M94C9, isolated from the soil of the Snow Antarctic Island [6]. As with other *Rhodotorula* species, the strain M94C9 is recognized as a polyextremotolerant yeast, exhibiting characteristics such as red colonies, growth adaptability to cold climates, and tolerance to high osmolarity and low nutrient availability [6]. The study aimed to investigate the composition of the mitochondrial respiratory chain of *R. mucilaginosa* M94C9, the presence of respiratory supercomplexes, and the kinetics of the respiratory enzymes. 

## 2. Materials and Methods

### 2.1. Strains and Growth Conditions

*R. mucilaginosa* M94C9 was maintained in 25% glycerol (*v*/*v*) at −70 °C and recovered in Yeast Peptone Dextrose Agar (YPD-agar) (0.5% yeast extract, 0.25% bactopeptone, 0.5% glucose, and 2% agar). Cells were cultured in 50 mL of YPD medium for 24 h at 200 rpm and 28 °C, recovered by centrifugation at 3000× *g*, washed once with 50 mL of sterile distilled water, and suspended in sterile water (1 mL H_2_O per g wet weight, pre-cultured cells). The pre-cultured cells were used to inoculate 1 L of fresh YPD with 60 absorbance units (final optical density at 600 nm of 0.06), after which they were incubated at 28 °C, at 200 rpm for 24 h. The strain was donated by Baeza M. All reagents for this research were purchased from Sigma Aldrich St. Louis, MO, USA. Except when indicated.

### 2.2. Dry Weight Determination

Aliquots of cell suspension (1.5–3 mL) were collected and centrifuged in pre-weighed tubes at 16,000× *g* in a tabletop microfuge. Then, the tubes were placed in an oven at 70 °C. After 72 h, the weight of the empty tubes was subtracted from the weight of the tubes containing the dry sample [7]. 

### 2.3. Glucose Determination

Aliquots of 1.5 mL were withdrawn at the indicated times and centrifuged for 1 min at 16,000× *g* in a tabletop microfuge. The supernatant was recovered and used for the determination of glucose using a colorimetric kit based on the activity of glucose oxidase (Glucose-TR, Spinreact, Girona, Spain).

### 2.4. Oxygen Consumption

Respiratory measurements of intact and permeabilized cells were carried out in 1.5 mL of air-saturated KME (120 mM KCl, 50 mM MOPS, 0.5 mM EGTA) buffer supplemented with 0.2 M trehalose, pH 7.0. Trehalose in the buffer was used to stabilize the proteins in the cells during the permeabilization process. Oxygen consumption was assayed in the absence of glucose; cells have a large endogenous reserve of glycogen. To identify the two terminal oxidases found in fungal mitochondria, two inhibitors were tested, 1 mM potassium cyanide (KCN) and 5 µM n-octyl gallate (nOG), which interact with the cytochrome c oxidase and the alternative oxidase (AOX), respectively. Oxygen consumption was determined using a Clark-type oxygen electrode [8]. 

### 2.5. Cell Permeabilization

Plasma membrane permeabilization was achieved by incubating 20 µL of cell suspension (1:1) in 1.5 mL of KME in the oximeter chamber plus a final concentration of 0.08–0.12% of digitonin. A decrease in oxygen consumption indicated cell permeabilization [8,9]. 

### 2.6. Mitochondria Isolation

Mitochondria were isolated according to Matuz-Mares [10] and Romero-Aguilar [11], and briefly described below. Rhodotorula cells were harvested by centrifugation at 1000× *g* for 5 min at 4 °C and washed twice with lysis buffer (20 mM Tris-HCl, 330 mM sucrose, 2 mM EDTA, 1 mM EGTA, and 100 mM KH_2_PO_4_, pH 7.4). For cell disruption, the biomass was resuspended following the ratio 1 g: 1 mL (wet weight/volume) with cold lysis buffer supplemented with 0.2% bovine serum albumin, 5 mM 2-mercaptoethanol, 1 mM phenylmethylsulfonyl fluoride, and protease inhibitor cocktail (Sigma Aldrich S8820-SIGMAFAST™, St. Louis, MO, USA). To achieve the disruption of the cells, 0.5 mL of glass beads (0.5 mm) was added to 0.5 mL of cell suspension. Mechanical disruption was performed in a Mini-Beadbeater with four cycles of 30 s of cell breakdown, followed by 2 min of incubation in an ice bath. Cytoplasmic extract and cell debris were separated from the glass beads by decantation and then centrifuged at 3000× *g* for 10 min. The supernatant was centrifuged at 12,000× *g* for 10 min. The mitochondrial pellet was suspended in a small volume of lysis buffer supplemented with 0.2% bovine serum albumin. The procedure was performed at 4 °C. The protein concentration was determined according to Lowry et al. [12]. 

### 2.7. Competition Plot

The competition plot was used to find out if two substrates (A, B) compete for the same site on the enzyme or if each one binds to different enzymes [13]. First, the initial concentrations of two substrates (A_0_, B_0_) are chosen such that they exhibit approximately the same specific activity. Second, the concentration of the substrates is varied according to the following equations: A = A_0_·(1 − *p*) and B = B_0_·*p*, where A_0_ and B_0_ are the initial concentrations of the substrates A and B that give the same specific activity, and *p* is a factor that changes, for each point, the concentrations of both substrates. Next, values of *p* = 0, 0.25, 0.5, 0.75, and 1, were used to calculate the concentrations of A and B at each point. The different values of *p* are calculated by adding a fixed increment of *p* = 0.25. For example, if *p* = 0, the concentration of A = A_0_ and B = 0; for *p* = 0.25, A = 0.75·A_0_ and B = 0.25·B_0_; for *p* = 0.5, A = 0.5·A_0_ and B = 0.5·B_0_; for *p* = 0.75, A = 0.25·A_0_ and B = 0.75·B_0_; finally, for *p* = 1, A = 0 and B = B_0_.

### 2.8. Bioinformatic Analysis

To find the genes that encode the subunits of the mitochondrial respiratory complexes in the *R. mucilaginosa*, a BLASTP search (https://blast.ncbi.nlm.nih.gov/Blast.cgi?PAGE=Proteins) was performed on the NCBI genome database of *R. mucilaginosa* KR using as queries the amino acid sequences of *Ustilago maydis* proteins on 1 June 2024. Three programs, DeepLoc [14], TargetP 2.0 [15], and WoLF PSORT [16], were used to predict the subcellular localization of the proteins. DeepLoc 2.0 predicts the subcellular localization of proteins in eukaryotic cells, such as fungal, mammalian, and plant cells. This program can differentiate between 10 different localizations, including mitochondria. TargetP-2.0 server predicts the presence of N-terminal sequences such as the signal peptide (SP) and the mitochondrial transit peptide (mTP). Importantly, this program predicts the cleavage site in the N-terminus of the protein. WoLF PSORT server also predicts the subcellular localization of fungal, plant, and animal proteins.

### 2.9. Blue Native-PAGE and in-Gel Catalytic Activity Assays

To solubilize the mitochondrial respiratory complexes of *R. mucilaginosa,* mitochondria were suspended to a final concentration of 10 mg/mL in 50 mM Bis-Tris and 500 mM 6-aminocaproic acid, pH 7.0 and digitonin (50% stock) was added drop by drop until a detergent/protein ratio of 3:1 was reached. At the same time, the mixture was gently stirred in an ice bath and incubated for 30 min in this condition. The sample was centrifuged at 100,000× *g* for 30 min at 4 °C and the supernatant recovered and supplemented with 10 µL of blue native buffer to a final concentration of 10% glycerol, 0.2% Coomassie Brilliant Blue G-250, and 20 mM 6-aminocaproic acid for blue native PAGE (BN-PAGE), and approximately 45–50 µg of protein was immediately loaded onto a linear polyacrylamide gradient gel (4–10%). The anode buffer solution contained 50 mM Bis-Tris/HCl, pH 7.0; the cathode buffer solution contained 50 mM tricine, 15 mM Bis-Tris, pH 7.0, and the anionic Coomassie dye (0.02 or 0.002%). BN-PAGE was run at 4 °C, at 35 V for 10 h. The molecular weight of the respiratory complexes and supercomplexes was determined by their electrophoretic mobility and in-gel catalytic activity using solubilized complexes of *U. maydis* as standards [10].

The in-gel activities were performed as described by Matuz-Mares et al. [10]. NADH dehydrogenase activity was assayed at 25 °C in a buffer containing 10 mM Tris/HCl, pH 7.4, 1.2 mM methylthiazolyldiphenyl tetrazolium bromide (MTT), and 1 mM NADH. Succinate dehydrogenase activity was assayed in 10 mM K_2_HPO_4_, pH 7.4, 10 mM succinate, 0.2 phenazine methosulfate (PMS), and 5 mM EDTA. The reaction was stopped with the fixing solution [50% methanol, 10% acetic acid (*v*/*v*)]. To measure the cytochrome c oxidase activity, the gel was incubated in 50 mM K_2_HPO_4_, pH 7.2, 4.7 mM 3,3′ diaminobenzidine tetrahydrochloride (DAB), and 16 µM horse heart cytochrome c. The reaction was stopped with the fixing solution. ATP-synthase was assayed in 50 mM glycine pH 8.0 (adjusted with triethanolamine), 10 mM MgCl_2_, 0.15% Pb(ClO_4_)_2_, and 5 mM Mg-ATP pH 7.0. After 60 min to 12 h incubation in the ATP mixture, the reaction was stopped with 50% methanol, and the gel was transferred to water and scanned against a dark background [10].

### 2.10. 2D SDS-PAGE

Two-dimensional tricine-SDS-polyacrylamide gel electrophoresis (2D SDS-PAGE) was performed according to Schägger et al. 1994 [17]. After BN-PAGE, proteins in a gel lane were separated by 2D tricine-SDS-PAGE on a 14% polyacrylamide gel under denaturing conditions. After the run, proteins were stained with Coomassie^©^ Brilliant Blue R-125.

### 2.11. Statistical Analysis

Statistical analysis of data was performed in GraphPad Prism V10. A two-way *t*-Student test was used to analyze the data. A statistically significant difference was defined for a *p* ≤ 0.05.

## 3. Results

### 3.1. Bioinformatic Results

Bioinformatic analysis revealed the presence of four classical respiratory complexes (I, II, III, and IV) and the ATP synthase (Complex V) in mitochondria of *R. mucilaginosa*. Additionally, several alternative components were identified, including an alternative oxidase, glyceraldehyde 3-phosphate dehydrogenase, five NADH dehydrogenases, and a lactate cytochrome b2 dehydrogenase. As detailed in Table 1 and Supplementary Materials (S1), Complex I (CI) or NADH dehydrogenase comprises at least 37 subunits in *R. mucilaginosa*: 7 of mitochondrial origin and 30 of nuclear origin. The number of subunits of the CI of *R. mucilaginosa* is like that found in *Neurospora crassa* (39 subunits), *Yarrowia lipolytica* (40 subunits), *Pichia pastoris* (41 subunits), and bovine (45 subunits) [18,19,20,21]. The molecular mass of *R. mucilaginosa* CI is close to 1 MDa (0.94 MDa) if the processing of proteins by mitochondria is considered. Complex II or succinate dehydrogenase comprises four subunits encoded by nuclear DNA. Furthermore, a fumarate reductase subunit was predicted in *R. mucilaginosa*, although current prediction methods placed this protein outside the mitochondria. A definitive experimental investigation is required to determine its subcellular localization and its potential to form a functional complex with fumarate reductase activity. The apparent molecular mass of CII is 123 kDa, consistent with literature findings [10]. Complex III consists of 10 subunits, one of mitochondrial origin and the remaining nine of nuclear origin. Given its function as an obligatory dimer, a predicted molecular mass of 548 kDa is attributed to the functional protein. Complex IV is composed of 11 subunits: 3 of mitochondrial origin and 8 of nuclear origin with a molecular mass of 208 kDa. Except for the Cox4 subunit, all other subunits are predicted to have mitochondrial localization (Table 1). Complex V is characterized by 16 distinct subunits, 3 of mitochondrial origin and 13 of nuclear origin. Considering the stoichiometry of 3*α*, 3*β*, and 10 *c* subunits, the molecular mass of CV is determined to be 607 kDa. In addition to the classical respiratory complexes, the bioinformatics analysis identified additional mitochondrial redox enzymes, such as glycerol 3-phosphate dehydrogenase (KAG0655011.1). Together with two cytosolic enzymes, it forms the glycerol 3-phosphate shuttle, facilitating the transfer of electrons from cytosolic NADH to the pool of quinones in the inner mitochondrial membrane. Five small-sized NADH dehydrogenases are also predicted with the smallest dehydrogenase (KAG0663538.1) likely not associated with mitochondria. Another important enzyme characteristic of most fungi is the alternative oxidase. As expected, a single gene for this enzyme was found in the genome of *R. mucilaginosa*. Additionally, a gene coding for a lactate cytochrome b2 dehydrogenase was also identified (KAG0667662.1) and is predicted to have mitochondrial localization.

### 3.2. Oxygen Consumption by Intact Cells: The Cytochrome c Oxidase and the AOX Are the Terminal Oxidases

**Figure 1:**
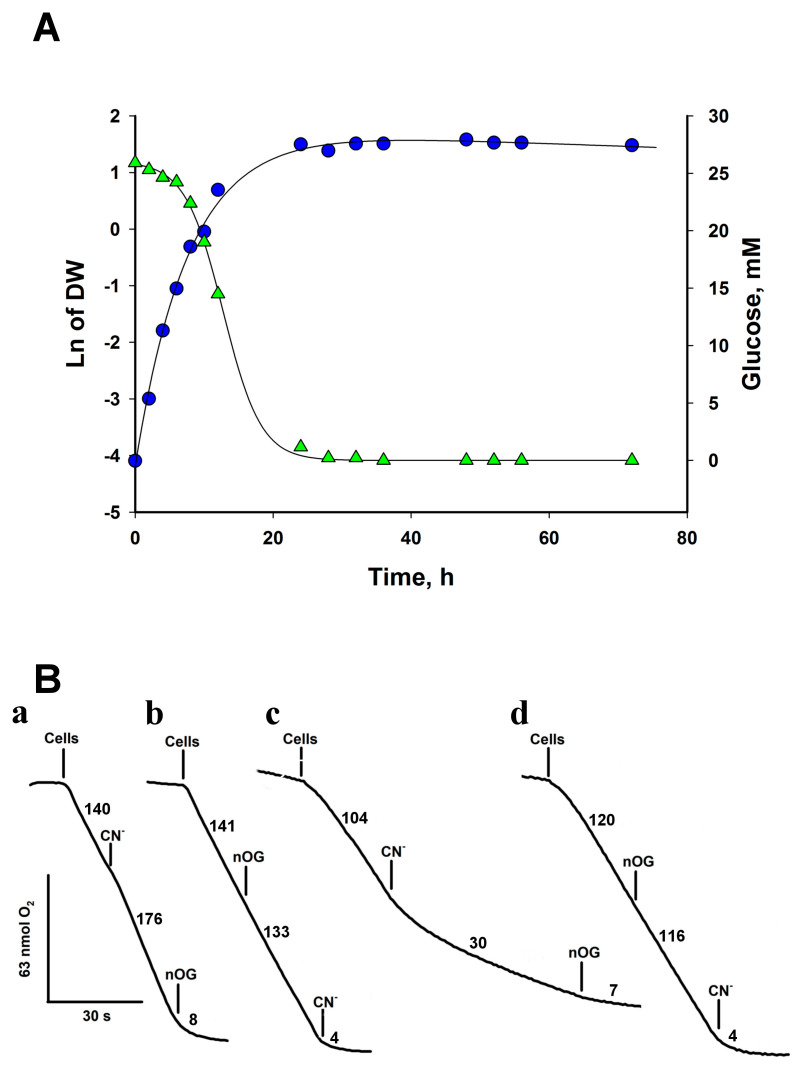
Growth curve and oxygen consumption by *R. mucilaginosa* cells. (**A**) Cells were grown in YPD for 72 h and aliquots were withdrawn and centrifuged at the indicated times to determine dry weight and glucose. Cells grew with a doubling time of 1.59 ± 0.15 h. (**B**) Oxygen consumption by cells. Respiratory traces of cells cultured in YPD for 24 h at 28 °C (a and b) and 6 h at 28 °C (c and d). The addition of nOG (5 µM) or KCN (1 mM) is indicated on the traces. Numbers indicate the rate of oxygen consumption in nmol·min^−1^. Representative data from at least four independent experiments.

As a first step in studying the energy metabolism of *Rhodotorula,* we measured oxygen consumption in whole cells obtained from 6- and 24-h YPD cultures. To study the capacity of AOX in cells from exponential and stationary growth phases, we harvested the cells at 6 h of growth when they were in the middle of the exponential phase, and there was enough biomass to carry out the experiments, and at 24 h, when the cells were at the beginning of the stationary phase (Figure 1A). The basal initial rate of O_2_ uptake by cells grown for 24 h in the culture medium was 102 ± 15 nmol min^−1^·mg dry weight^−1^ (Figure 1A, Table 2). Similar values for specific oxygen consumption rates have been reported for *S. cerevisiae* [22] and *Y. lipolytica* [23], although the rates in *Y. lipolytica* can be as high as 670 nmol min^−1^·mg. dry weight^−1^ [23]. On the other hand, the addition of 1 mM KCN produced activation (Figure 1B(a)) of oxygen consumption. The cyanide-resistant respiratory activity (117 ± 23 nmol min^−1^·mg dry weight^−1^) in cells from 24 h was inhibited by 5 µM of nOG, a classic inhibitor of the AOX from protozoa [24], plants [25], and fungi [26] (Figure 1B(b)). This result suggests that *R. mucilaginosa* mitochondria contain two terminal oxidases, the cytochrome c oxidase, inhibited by cyanide, and the cyanide-insensitive alternative oxidase (Figure 1B).

The expression of AOX in *Y. lipolytica* [27] and *U. maydis* [28] changes with the growth phase. In the exponential phase, the concentration of AOX in mitochondria is low, but during the stationary phase, its expression increases [28]. To test whether *R. mucilaginosa* followed this pattern, the cells were harvested in the middle of the exponential phase (6 h of growth, Figure 1A) and the effect of inhibitors on oxygen consumption was studied to determine the relative capacity of AOX in mitochondria. Figure 1B(c) shows that CN^−^ produced a significant inhibition of respiration (70%), indicating that mitochondria in cells from the exponential phase have less AOX than in the stationary phase (Figure 1B(a)). In the presence of CN^−^, nOG inhibited respiration in any conditions (Figure 1B(a,d). On the other hand, if nOG is added first, the O_2_ consumption rate of the cells decreased by only 4%, indicating that the cytochrome chain is not inhibited by 5 µM nOG and that inhibition of AOX hardly affected the respiratory activity of cells (Figure 1B(b,d)). As expected, in the presence of the two inhibitors, O_2_ consumption was strongly inhibited (Figure 1B). The data indicate that the mitochondria of *R. mucilaginosa* have the cytochrome c oxidase and the AOX as terminal oxidases and that the activity of AOX is higher in the stationary phase (Figure 1B(c)).

### 3.3. Respiratory Activities in Permeabilized Cells: Mitochondria Contain an Alternative NADPH Dehydrogenase

**Figure 2:**
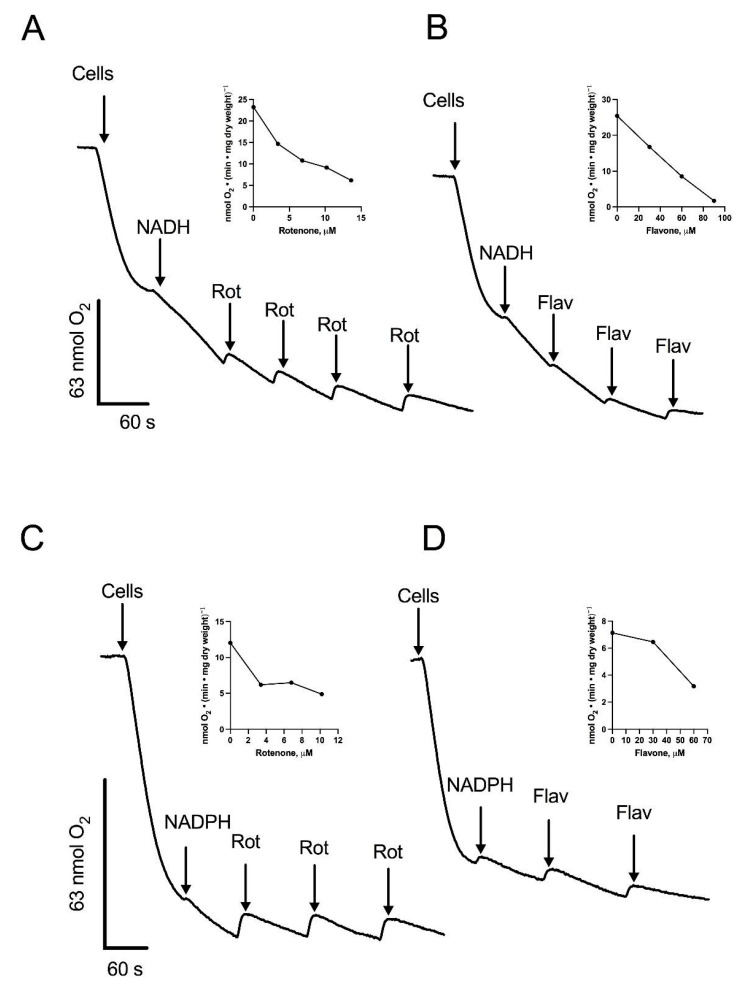
Stimulation of oxygen consumption by NADH or NADPH in permeabilized cells. Cells were permeabilized with digitonin and the respiratory activity was stimulated by the addition of NADH or NADPH. Aliquots of a solution containing rotenone (Rot) or flavone (Flav) were added at the indicated times during the assay. Oxygen consumption supported by NADH and inhibited by Rot (**A**) or Flav (**B**). Oxygen consumption supported by NADPH and inhibited by Rot (**C**) or Flav (**D**). Arrows indicate additions. The inserts depict the inhibition of respiratory activity by increasing concentrations of Rot (**A**,**C**) or Flav (**B**,**D**). Representative data from four independent experiments are shown.

**Figure 3:**
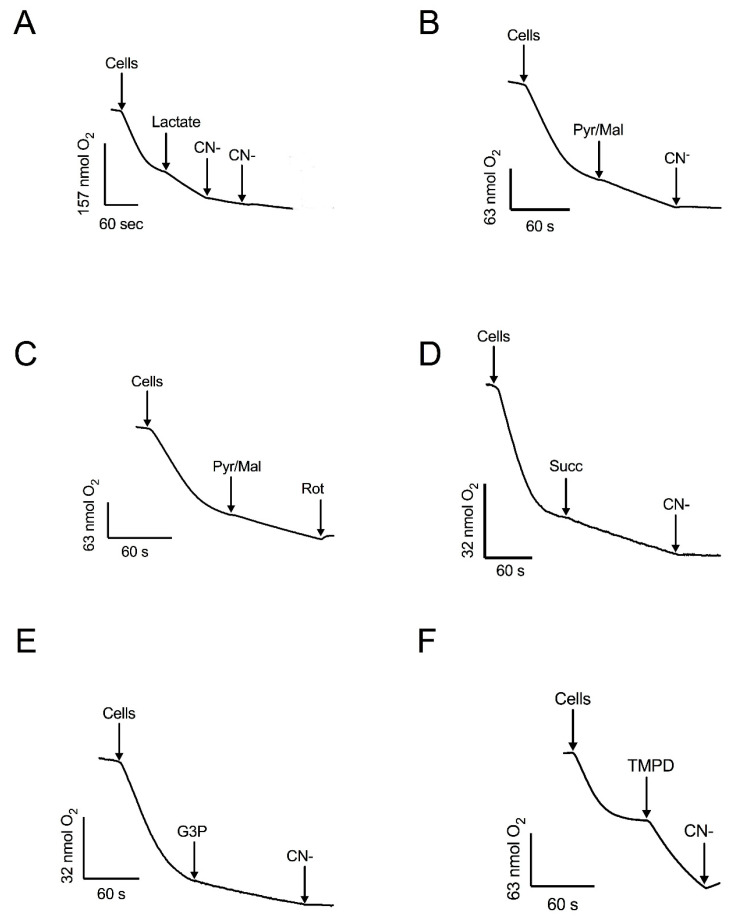
The effect of mitochondrial substrates and inhibitors of oxygen consumption by permeabilized cells. Respiratory activity was stimulated by: (**A**) DL-lactate and inhibited by CN^−^. (**B**) Pyruvate-malate (Pyr/Mal) and inhibited by CN^−^. (**C**) Pyr/Mal and inhibited by rotenone (Rot). (**D**) succinate (Succ) and inhibited by CN^−^. (**E**) Glycerol 3-phosphate (G3P) and inhibited by CN^−^. (**F**) TMPD and inhibition by CN^−^. Arrows indicate additions. Representative data from four independent experiments are shown.

To further characterize the components of the respiratory chain in *R. mucilaginosa* mitochondria, we utilized 0.12% (*w*/*v*) digitonin to permeabilize the yeast cells plasma membrane, providing direct access to the mitochondria. One of the main advantages of the permeabilization technique is the preservation of the internal structures of the cells, including the interactions of mitochondria with other organelles. Additionally, plasma membrane permeabilization allows the free diffusion of mitochondrial substrates and inhibitors, facilitating the study of the respiratory chain function [29]. Upon permeabilization, oxygen consumption was measured at pH 7.0, since *S. cerevisiae* [30], *N. crassa* [31], and *U. maydis* [32] all have intracellular pH values close to 7 (6.99–7.30, 7.19, and 7.03, respectively). In the presence of digitonin, the respiratory activity of cells decayed with time due to the release of mitochondrial substrates into the respiration buffer (Figure 2). The addition of 1 mM NADH restored the respiratory activity of the cells, indicating the transfer of electrons from NADH into the electron transport chain (ETC), and suggesting the oxidation of NADH by an external NADH dehydrogenase (Figure 2A). Notably, rotenone, a complex I inhibitor, produced a decrease in oxygen consumption (Figure 2A). In line with the presence of an external NADH dehydrogenase, respiratory activity was inhibited by flavone, an inhibitor of alternative NADH dehydrogenases (Figure 2B) [33,34]. Our bioinformatics analysis showed the presence of four putative NAD(P)H dehydrogenases and one lactate cytochrome b2 dehydrogenase. Consequently, we investigated NADPH and lactate as potential substrates (Figure 2C,D and Figure 3A). As shown in Figure 2C,D and Figure 3A, respiration was stimulated by NADPH and lactate, respectively. Interestingly, oxygen consumption in the presence of lactate was not fully inhibited by cyanide (Figure 3A). Flavone and rotenone inhibited the respiratory activity of permeabilized cells incubated in the presence of NADPH, with the inhibition pattern differing from that observed with NADH, suggesting the presence of two different enzymes (Figure 2A–D).

**Figure 4:**
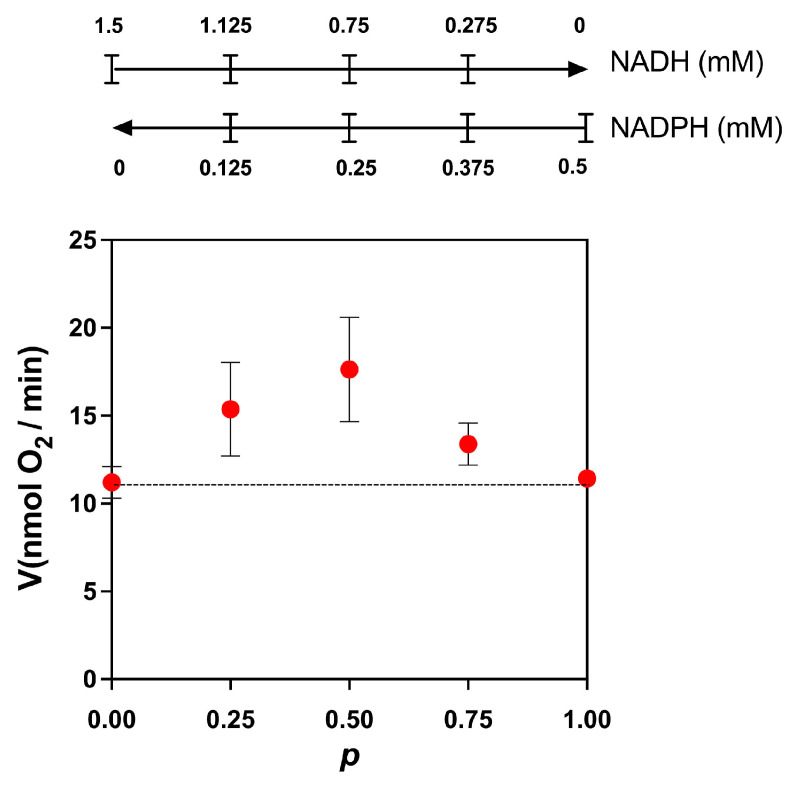
Competition plot for NADH and NADPH. The total rate of reaction was determined by stimulating the oxygen consumption by adding mixtures of NADPH and NADH, as indicated above in the figure. The initial concentration of NADH was 1.5 mM. At this concentration, the rate of reaction in the absence of NADPH was 11.2 ± 0.9 nmol O_2_/min. For NADPH, the initial concentration was 0.5 mM, giving a rate of 11.4 ± 0.3 nmol O_2_/min. When the curve is above the horizontal line (dotted line), the substrates act at different sites (enzymes). Data are the mean and standard deviation of three independent experiments.

Next, we used the competition plot to find out if these two substrates compete for the same site on the enzyme or if each one is oxidized by different dehydrogenases. In this plot, the initial concentrations of NADH and NADPH were chosen such that we obtained approximately the same specific activity. The two concentrations were selected by a trial-and-error process. Next, each concentration was varied according to the following equations: NADH = NADH_0_·(1 − *p*) and NADPH = NADPH_0_·*p*, where NADH_0_ and NADPH_0_ are the initial concentrations of the substrates and *p* is a factor that changes the concentrations of both substrates. In our experiment, we selected increments of 0.25 to calculate the values of *p* (*p* = 0, 0.25, 0.5, 0.75, and 1) to calculate the concentrations of NADH and NADPH at each point. The only requirement for constructing the competition plot is that both substrates produce approximately the same specific activity. According to the theory [13], when a horizontal line is obtained, the two substrates compete for the same site, and when the curve is above the horizontal line, the substrates act at different sites (enzymes). As shown in Figure 4, the competition plot indicates the presence of two enzymes, one specific for NADH and another for NADPH.

Respiration was also supported by pyruvate-malate (Figure 3B, C), which depends on the production of NADH in the mitochondrial matrix. In the presence of these substrates, oxygen consumption was fully inhibited by rotenone or cyanide (Figure 2 and Figure 3). Additionally, mitochondria in permeabilized cells also responded to succinate (Figure 3D) and glycerol-3-phosphate (Figure 3E), indicating the presence of functional succinate dehydrogenase and the glycerol-3-phosphate shuttle. Cyanide inhibited the respiratory activity with these substrates. The highest stimulation of the respiratory activity was obtained in the presence of TMPD, a compound that feeds into the electron transport chain at the level of complex IV (Figure 3F). Taken together, the experiments with permeabilized cells, utilizing various substrates and inhibitors, indicate that mitochondria of *R. mucilaginosa* contain, in addition to the classic respiratory complexes I, II, III, and IV, five additional components: the glycerol 3 phosphate dehydrogenase, external NADH and NADPH dehydrogenases, lactate cytochrome b2 dehydrogenase, and the alternative oxidase.

Next, we investigated the impact of varying concentrations of mitochondrial substrates on respiratory activity. The rate of oxygen consumption in permeabilized cells was fitted to the Michaelis–Menten kinetics with NADH, NADPH, succinate, and lactate as substrates. Two key parameters, K_0.5_ and V_max_, were calculated through curve fitting of the kinetic data. The values of K_0.5_ in permeabilized cells for NADH, NADPH, succinate, and lactate were 61 ± 42, 149 ± 37, 108 ± 26, and 41 ± 28 μM, respectively. This suggests a higher apparent affinity of *R. mucilaginosa* mitochondria for NADH and lactate, based on K_0.5_. Notably, with NADH, oxygen consumption showed the highest activity with a V_max_ of 28 ± 6 nmol min^−1^ mg dry weight^−1^ followed by NADPH (21 ± 4 nmol min^−1^ mg dry weight^−1^), DL-lactate (17 ± 1 nmol min^−1^ mg dry weight ^−1^), and succinate (10 ± 1 nmol min^−1^ mg dry weight^−1^) (Table 3).

### 3.4. Respiratory Activities in Isolated Mitochondria

It has been reported that the affinities of components of the respiratory chain in mitochondria and permeabilized cells can differ, partly because the interactions of the mitochondria with other organelles are maintained in the permeabilized cells and partly because the structure of mitochondria can be damaged during isolation [35,36]. Therefore, we isolated mitochondria through differential centrifugation and studied the saturation kinetics of the different respiratory substrates. The kinetics for each substrate was also fitted by the Michaelis–Menten model. The apparent affinity was approximately the same for all substrates, that is, all of them are in the micromolar range (Table 3). Furthermore, except for NADPH, no statistically significant differences were observed between the affinities of the enzymes in permeabilized cells and mitochondria. Similar to permeabilized cells, the maximum oxygen consumption rate in mitochondria was obtained with NADH [299 ± 82 nmol · min^−1^ · mg protein^−1^] while the smallest with succinate [52 ± 6 nmol · min ^−1^ · mg protein^−1^]. The data show that NADPH and lactate cytochrome b2 dehydrogenases are components of the respiratory chain of *R. mucilaginosa* mitochondria; in addition, the K_0.5_ values for the dehydrogenases were in the micromolar range in mitochondria and permeabilized cells (Table 3). Here, K_0.5_ was preferred over K_m_ because the oxygen consumption induced by the substrate is the result of several enzymes in the electron transport chain. In contrast, K_m_ is used for isolated enzymes that follow the Michaelis–Menten kinetics.

### 3.5. Respiratory Components of R. mucilaginosa Are Organized in Complexes and Supercomplexes

**Figure 5:**
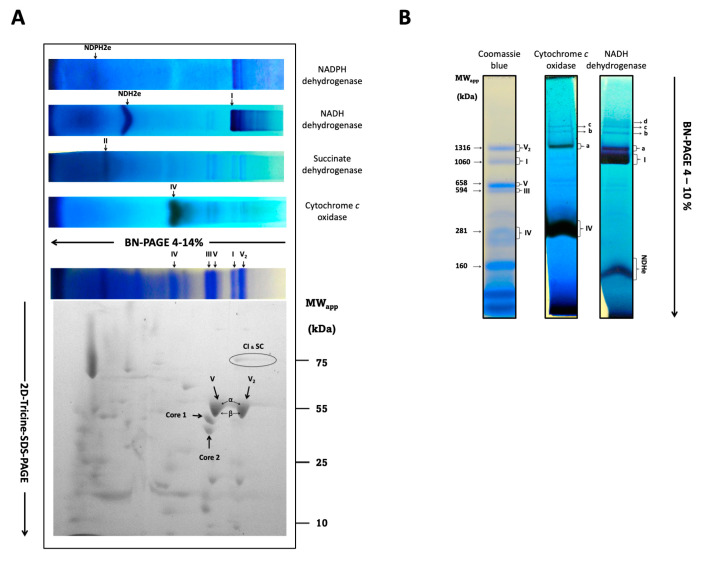
In-gel activity of the *R. mucilaginosa* respiratory complexes. Respiratory complexes and supercomplexes were solubilized with digitonin at a 3:1 ratio and subjected to BN-PAGE (CI to CIV). (**A**) Activity in native gel strips for alternative NADH and NADPH dehydrogenases, the respiratory complexes II, IV, the Coomassie stain, and I. The activity of NADH dehydrogenase also shows the presence of CI supercomplexes at ≈1254, ≈1691, ≈1937, and ≈2046 kDa. For details see main text. The presence of CV_2_, CIII_2_, CIV, and the supercomplex composed of CIV and CI are shown in the Coomassie-stained gel. Respiratory complexes subunits were solved in a 2D-tricine-SDS-PAGE. The presence of the 78 kDa (NUAM) subunit of CI, the 43 (QCR2), and 50 (QCR1) kDa subunits of CIII, and the *α* (46.5) and *β* (59) kDa subunits of CV and CV_2_ are indicated. The *α* and *β* subunits were not resolved. (**B**) In-gel activity showing the supercomplexes CI and CIV.

**Figure 6:**
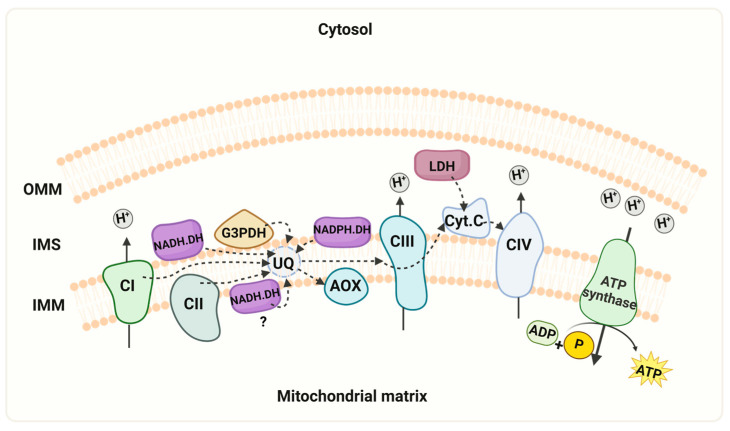
Schematic representation of the mitochondrial respiratory chain. The model depicts the mitochondrial respiratory chain of *R. mucilaginosa,* containing the four multi-subunit complexes (CI, II, III, and IV), two mobile components (ubiquinone and cytochrome c), and the ATP synthase. Additionally, alternative pathways of NADH dehydrogenases (NADH.DH), NADPH dehydrogenase (NADPH.DH), lactate dehydrogenase (LDH), and the presence of the alternative oxidase (AOX) are illustrated. In the experimental conditions described here, we did not observe activity of the internal alternative NADH dehydrogenase. However, we cannot discard that the enzyme can be expressed under different growth conditions. Key features include the mitochondrial intermembrane space (IMS), outer mitochondrial membrane (OMM), inner mitochondrial membrane (IMM), cytochrome c (Cyt c), ubiquinone (UQ). “Created with BioRender.com”. Agreement number ZU26JCFNMO.

Recently, the respiratory complexes I, II, IV, and V, and the monomer and dimer of CV of this microorganism were identified by BN-PAGE of mitochondria solubilized with digitonin, followed by a careful proteomic study [5]. However, there was no mention of respiratory supercomplexes or the activity of the ATP synthase monomers and dimers in the BN-PAGE [5]. Therefore, we investigated the formation of respiratory supercomplexes in *R. mucilaginosa*. Mitochondrial proteins were solubilized with digitonin at a 3:1 digitonin/protein ratio and resolved by BN-PAGE. As shown in Figure 5, mitochondria of *R. mucilaginosa* contain the free respiratory complexes I, II, III_2_, and IV. Molecular masses for the free complexes calculated were 937 (CI), 208 (CIV), 548 (CIII_2_), 123 (CII), and 607 (CV) kDa. In addition to the free respiratory complexes, supercomplexes containing complexes I and IV were detected on the gel (Figure 5B). Remarkably, complex I can utilize NADPH as a substrate both in its free form and as part of the supercomplexes (Figure 5A,B). It should be noted that the molecular weights of the *R. mucilaginosa* and *U. maydis* free complexes and supercomplexes were very similar [10]. In addition to the classic respiratory complexes, we found two alternative NAD(P)H dehydrogenases below complex I in the BN-PAGE gel with molecular masses around 119 (NADPH dehydrogenase) and 154 kDa (NADH dehydrogenase) (Figure 6). Finally, ATPase activity associated to CV was not detected on the BN-PAGE, but ATPase bands on the SDS-PAGE were observed at 600 and 1200 kDa, indicating the presence of the monomeric and dimeric forms of the ATP synthase (Figure 5A,B). 

To find the characteristic subunits of each respiratory complex, we subjected the lane of the gel with the complexes and supercomplexes to second-dimension electrophoresis in a gel with SDS as denaturing agent (2D-Tricine-SDS-PAGE). Densitometric analysis of the 78 kDa band of complex I (NUAM) indicate that this complex has a higher concentration in its free form than when it is linked to supercomplexes. Unexpectedly, the *α* and *β* subunits of the monomeric and dimeric ATP synthase migrated as a single and deformed band (Figure 5A), despite the difference in molecular masses of the two subunits. The intensity of both spots was similar, suggesting that the monomeric and dimeric forms of the ATP synthase in *R. mucilaginosa* mitochondria were present in comparable amounts (Figure 5A and Supplementary Materials S2). Complex III was located beneath the monomeric ATPase (Figure 5A). Complex IV subunits can be found below complex III.

## 4. Discussion

Early investigations in *Rhodotorula gracilis* revealed the presence of a cyanide-resistant electron transport chain [37,38], now identified as the mitochondrial alternative oxidase. Interestingly, these studies found activation of respiratory activity by cyanide in cells and the loss of the AOX activity during subcellular fractions preparation [37,38], pointing to the high sensitivity of the AOX to stressful environmental conditions. However, in contrast to our mitochondrial preparation with no AOX activity, the isolation of mitochondria with an active AOX has been reported recently [5]. Difference spectra of diphenylamine-treated cells (to inhibit carotenoid formation) also showed the presence of type *a*, *b*, and *c* cytochromes, suggesting the presence of complexes III and IV [37,38]. Here, we aimed to further characterize the mitochondrial respiratory chain of *R. mucilaginosa* M94C9 using a bioinformatic analysis as an initial step, followed by oxygen consumption experiments in intact and permeabilized cells and mitochondria to stimulate or inhibit the respiratory enzymes with specific substrates and inhibitors.

The bioinformatic analysis on the *R. mucilaginosa* KR database showed the presence of genes coding for the subunits of complexes I, II, III, IV, and V, a single gene for the AOX, five alternative NAD(P)H dehydrogenases, the glycerol 3-phosphate dehydrogenase, and the lactate cytochrome b2 dehydrogenase. The study of the respiratory activity of intact cells revealed the presence of complex IV and the AOX. In cells grown for 24 h in a rich medium, respiration showed stimulation upon the addition of KCN. As expected, upon the consecutive addition of nOG, oxygen consumption was highly depressed. The impact of KCN on *R. mucilaginosa* respiratory activity exhibited a dependence on the growth phase of the cells. During the middle exponential phase (6 h of culture, Figure 1A), inhibition of the respiratory activity by KCN was observed. However, at longer times oxygen consumption was stimulated, indicating a higher concentration of AOX in mitochondria. Interestingly, similar results were obtained in other yeasts such as *R. gracilis* [37,38], *Y. lipolytica* [27,39]*,* and *U. maydis* [8,28]. In agreement with another report [22], *R. mucilaginosa* displayed a higher basal respiratory activity in the exponential phase (166 ± 18 nmol·min^−1^·mg dry weight^−1^), which decreased to 102 ± 15 nmol·min^−1^·mg dry weight^−1^ when cells reached the early stationary phase (24 h), and a further decrease at 72 h (59 ± 2 nmol·min^−1^·mg dry weight^−1^), indicating a lower respiratory capacity when cells transit from the exponential phase to the stationary phase. Taken together, the results suggest the presence of non-heme cyanide-resistant alternative oxidase (AOX) and the classic cytochrome c oxidase or complex IV in the respiratory chain.

AOX has many functions, including heat production for the release of volatile compounds to attract insect pollinators [40], protection against oxidative stress [41], and various stressors [42], participation in pathogenicity [41,43], involvement in the production of secondary metabolites [44]. From a metabolic point of view, AOX contributes to the flexibility of carbon and energy metabolism by providing a mechanism to relax the tight coupling between the respiratory chain and the synthesis of ATP. In *Y. lipolytica* the AOX gene is upregulated during stress conditions [45]. Although AOX activity was increased in *R. mucilaginosa* M94C9 after 24 h of cell culture, the underlying mechanism for their expression and its possible participation in the response against oxidative stress remains unclear. It is necessary to conduct more studies on this alternative element.

Next, we assessed the activities of respiratory enzymes in both permeabilized cells and isolated mitochondria of *R. mucilaginosa.* The observed differences in the inhibition patterns between flavone and rotenone and the results of the competition plot indicate that mitochondria of *R. mucilaginosa* possess external NADH and NADPH dehydrogenases (NDH2e, NDPH2e) (Figure 2 and Figure 4). External alternative NADPH dehydrogenases are found frequently in plant and fungal mitochondria [46,47,48,49]. Further work is required to test the role of NADPH dehydrogenases in *R. mucilaginosa* metabolism.

Oxygen consumption also was stimulated by glycerol 3-phosphate, succinate, pyruvate-malate, and lactate, indicating the presence of glycerol-3-phosphate dehydrogenase, complexes II and I, and lactate cytochrome b2 dehydrogenase, respectively (Figure 3). When using the first three substrates, respiration was fully inhibited by cyanide, suggesting the loss of AOX activity during the permeabilization of cells. This result agrees with the reported loss of AOX activity in subcellular fractions of *R. gracilis* [37]. However, in the presence of DL-lactate, cyanide produced a partial inhibition of respiratory activity, which can be explained by the transfer of electrons from lactate to oxygen [50]. In contrast to the resistance of the external NAD(P)H dehydrogenases to rotenone, low concentrations of this inhibitor stopped oxygen consumption in the presence of pyruvate-malate, suggesting that the respiratory activity with pyruvate-malate was supported by complex I. The full inhibition of respiratory activity by low concentrations of rotenone indicates the absence of an internal alternative NADH dehydrogenase, emphasizing the importance of complex I in *R. mucilaginosa* M94C9 for the oxidation of NADH produced by the Krebs cycle and other mitochondrial matrix reactions. Considering that the binding sites of NDH2e and the glycerol 3 phosphate dehydrogenase face the mitochondrial intermembrane space, these enzymes likely play a role in the oxidation of cytosolic NADH generated by the glycolytic pathway and other biosynthetic reactions [51,52].

The K_0.5_ values for NADPH, NADH, succinate, and lactate indicate that *R. mucilaginosa* M94C9 cells exhibit apparent affinities in the micromolar range. Interestingly, similar K_0.5_ values were obtained in both permeabilized cells and isolated mitochondria (Table 3), indicating that the disruption of the mitochondrial contacts with other organelles did not affect the kinetic parameters of the enzymes.

The presence of complexes I, II, III, IV, V, and two alternative elements, NADH and NADPH dehydrogenases, were visualized by BN-PAGE and the SDS-PAGE. Moreover, we show for the first time the presence of supercomplexes in *R. mucilaginosa* M94C9 mitochondria, which are similar to those reported for *U. maydis* [10]*, Y. lipolytica* [17]*, D. hansenii* [53]*,* and *N. crassa* [54]. Respiratory supercomplexes may facilitate more efficient electron transfer between their constituent complexes, thereby enhancing the overall efficacy of the electron transport chain [55]. Although there is no structural channeling, the close association between the complexes within the supercomplexes reduces the diffusion distance for electron-carrying molecules, increasing the electron flow through the chain. Additionally, by promoting the efficient transfer of electrons within the supercomplexes, these structures may help mitigate the production of reactive oxygen species [56], which are detrimental by-products of mitochondrial metabolism. A further potential role of the supercomplexes is the stabilization of individual complexes, particularly complex I [57], shielding them from degradation or denaturation and ensuring their proper functioning.

Regarding *R. mucilaginosa,* the experimental molecular masses of respiratory complexes calculated using the *U. maydis* proteins as markers and those obtained from the *R. mucilaginosa* genome are in close agreement. Free complex I migrated with a molecular mass of 937 kDa, CII 130 kDa, CIII 548 kDa, CIV 208 kDa, CV_1_ 600 kDa, and CV_2_ 1200 kDa, which also agree, within the experimental variation, with those reported by Castañeda-Tamez. (CI 1000 kDa, CII 100 kDa, CIII 580 kDa, CIV 200 kDa, CV_1_ 580, and CV_2_ 1130 kDa) [5]. Additionally, we obtained molecular masses of 154 and 119 kDa for the alternative NADH and NADPH dehydrogenases, respectively, suggesting a dimeric structure for both enzymes. The presence of mitochondrial NADPH dehydrogenases is found in fungi and plants [46,47,48,49]. The deletion of the coding gene for the NADPH dehydrogenase in *Arabidopsis thaliana* reduced both the vegetative growth and the concentration of Krebs cycle intermediates, suggesting a secondary role for this enzyme in the growth and the homeostasis of NADH/NADPH [58]. In addition to the single respiratory complexes, several supercomplexes containing CI and CIV were observed. Using the mitochondrial proteins of *U. maydis* as molecular weight markers, four supercomplexes were predicted (Table 4). Based on the molecular weight of the NDH2e (Figure 5A,B) and the experimental molecular masses of the supercomplexes, we propose that the external NADH dehydrogenase might participate in the formation of supercomplex I_1_III_2_IV_2_NDHe2. Association of the external NADH dehydrogenase with CIII and CIV has been reported for *Y. lipolytica* [47]; however, this remains to be explored. On the other hand, similar supercomplexes have been reported in *U. maydis* [10]*, D. hansenii* [53], and *Y. lipolytica* [17] (Table 5). In contrast with the above microorganisms, the supercomplex I_1_III_2_ was absent in *R. mucilaginosa*. The densitometric analysis with ImageJ 1.54h (https://imagej.net/ij/) of the 78 kDa band in the SDS-PAGE gel revealed that 60% of complex I is found in the free form and nearly 40% corresponds to the I_1_IV_1_ supercomplex (band a) (Figure 5B and Supplementary Materials S2). Like in other fungi [9,21,45,53], CI is stable in its free form even after digitonin solubilization. Unlike *N. crassa* [54], the formation of CI dimer was not observed. Although we observed respiratory complex associations, the amount of proteins in bands b, c, and d were so scarce that they were not observed in the Coomassie-stained gel (Figure 5B). Conversely, the most abundant protein in the inner mitochondrial membrane was complex V, with 54% of the enzyme in the monomeric form and 46% as a dimer. For this calculation, we relied on the denaturing gel to analyze the oligomeric states of CV because it seems that the Coomassie in the BN-PAGE and/or the lead in the reaction mixture inhibited the activity of the ATPase. A similar proportion of ATP synthase was reported by Castañeda-Tamez 2024 [5] for cells grown for 24 h, although the proportion of V_1_ and V_2_ also depended on the carbon source and the growth phase; in lactate, the dimer was more abundant than the monomer [5]. In the SDS-PAGE, the *α* and *β* subunits of CV were not separated, remaining to be investigated. In summary, the respiratory chain of *R. mucilaginosa* follows very closely the general composition found in other fungal microorganisms. However, it remains to be investigated if the external NADH dehydrogenase participates in the formation of some supercomplexes.

Our results differ from those presented by Castañeda-Tamez et al. [5] in two aspects. First, in contrast to our results, the authors reported higher alternative oxidase activity in cells during the exponential growth compared to the stationary phase. This discrepancy may be attributed to differences in the designation of the exponential phase, as our analysis of their growth data in the semilog plot suggests that their exponential phase ended at 12 h, with cells exhibiting a large reduction in growth rate by 24 h. In our experiments, cells reached the stationary phase at 24 h, with the middle exponential phase occurring at 6 h (Figure 1A). Second, our respiratory activities in cells were approximately 20 times higher than those reported by Castañeda-Tamez et al., 2024 [5]. For instance, the basal respiratory activity in their cells grown for 24 h was close to 7 µmol O_2_ min^−1^ g dry weight^−1^ (if 1 nmol O_2_ = 2 natg O and 1 g dry weight = 5 g wet weight for *R. mucilaginosa* whereas we obtained 102 µmol O_2_ min^−1^ g dry weight ^−1^. We do not have a clear explanation for this large difference, but it may depend on the strains used in the two works. Nonetheless, our basal respiratory activity agrees with values reported for other yeasts [22,23,59]. Interestingly, despite the large difference in cellular oxygen consumption, the duplication times calculated from the points in the exponential phases were similar in both studies (1.38 h and 1.59 h, respectively).

## 5. Conclusions

*R. mucilaginosa*, a prominent species of the Rhodotorula genus, stands out for its highly significant carotenoid production, rendering it crucial in biotechnology. However, recent years have seen the emergence of Rhodotorula as a recognized yeast pathogen in humans [4,60,61]. This study makes a significant contribution to the understanding of the respiratory metabolism of *R. mucilaginosa.* Particularly, we present evidence of the capacity of complex I to use NADPH as a substrate, the activity of an NADPH dehydrogenase, and the presence of several respiratory supercomplexes in the extremotolerant strain *R. mucilaginosa* M94C9 isolated from the soil of the Snow Island in Antarctica [6]. As depicted in Figure 6, the ETC of *R. mucilaginosa* M94C9 contains an AOX, external NADH and NADPH dehydrogenases, the glycerol-3- phosphate dehydrogenase, and a lactate dehydrogenase. Notably, this repertoire extends beyond the classic respiratory complexes I, II, III, IV, and the ATP synthase (CV). However, we cannot discard the presence of an alternative NADH dehydrogenase facing the mitochondrial matrix under different experimental conditions (Figure 6), but further investigation is warranted to confirm this aspect. In conclusion, our findings shed light on the respiratory chain structure an activity of *R. mucilaginosa* M94C9 and the formation of supercomplexes, which contribute to the evolving status of *R. mucilaginosa* as a model organism (Figure 6).

## Figures and Tables

**Table 1:** Number of nuclear and mitochondrial genes encoding the components of the electron transport chain, and their putative molecular masses.

	CI	CII	CIII	CIV	CV
Mitochondrial encoded	7	0	1	3	3
Nuclear encoded	30	4	9	8	13
Molecular mass (kDa)	937.4	123.2	274	208.3	622.2
Protein/accession number			Molecular mass (kDa)		
Alternative oxidase KAG0654415.1			43.2		
Glycerol 3-P dehydrogenase KAG0655011.1			96.4		
Lactate cyt b2 dehydrogenase KAG0667662.1			57.4		
NAD(P)H dehydrogenases:					
KAG0665032.1			62.3		
KAG0656515.1			49.8		
KAG0656514.1			51.4		
KAG0663112.1			74.1		
KAG0663538.1			41.8		

**Table 2:** Respiratory activity in intact cells, permeabilized cells, and isolated mitochondria. Cells were grown for 24 h.

Respiratory Activity [nmol O_2_ min^−1^ mg Dry Weight ^−1^] *	Substrate (mM)
102 ± 15	Initial
117 ± 23	KCN
97 ± 18	nOG
Permeabilized cells *	
22.3 ± 5.9	NADH
14.4 ± 4.0	NADPH
11.6 ± 1.9	Succinate
13.9 ± 1.2	Lactate
6.9 ± 3.8	Pyruvate-malate
3.0 ± 1.2	Glycerol-3-phosphate
48.0 ± 23.9	TMPD
Isolated Mitochondria	
[nmol O_2_ min^−1^ mg protein^−1^]	
241.5 ± 29.7	NADH
192.9 ± 25.4	NADPH
53.6 ± 12. 2	Pyruvate-Malate
60.1 ± 10.9	Succinate
69.2 ± 1.1	Lactate
294.3 ± 81.1	TMPD

KCN, cyanide potassium. nOG, n-octyl gallate, NADH, Nicotinamide adenine dinucleotide reduced. NADPH, nicotinamide adenine dinucleotide phosphate reduced. TMPD, N, N, N′, N′ tetramethyl-p-phenylenediamine dihydrochloride. Cells were grown for 24 h and then were harvested and treated as indicated in the Materials and Methods section to permeabilize and obtain the mitochondria. * units of respiratory activity assayed in permeabilized and the intact cells.

**Table 3:** Kinetic parameters of mitochondrial enzyme activities. Kinetic parameters were obtained by using different concentrations of substrate to stimulate mitochondrial enzyme activity in permeabilized cells and isolated mitochondria.

	Condition/Units	Substrate
	Permeabilized cells	
K_0.5_ (μM)	V_max_ (nmol·min^−1^·mg dry weight ^−1^)	
61 ± 42	28 ± 6	NADH
149 ± 37 *	21 ± 4	NADPH
108 ± 26	10 ± 1	Succinate
41 ± 28	17 ± 1	DL-Lactate
Isolated Mitochondria		
K_0.5_ (µM)	V_max_ (nmol·min^−1^·mg protein^−1^)	
54 ± 31	299 ± 82	NADH
56 ± 16	206 ± 49	NADPH
64 ± 47	52 ± 6	Succinate
20 ± 13	73 ± 2	DL-Lactate

Data are representative of at least three independent experiments. * *p* < 0.05.

**Table 4:** Molecular masses and suggested stoichiometry for *R. mucilaginosa* respiratory supercomplexes.

Band	Apparent Mass (KDa)	Calculated Mass (KDa)	Tentative Stoichiometry
a	1254	1145	I_1_IV_1_
b	1691	1693	I_1_III_2_IV_1_
c	1937	1901	I_1_III_2_IV_2_
d	2046	2055 *	I_1_III_2_IV_1_X*

X*. This component could be NDH2e (154 kDa). Masses were calculated from apparent masses of constituent complexes.

**Table 5:** Molecular masses (kDa) of respiratory complexes of some fungi.

Complexes	*R. mucilaginosa*		*R. mucilaginosa* *	*U. maydis* **	*Y. lipolytica* ***
	Experimental	Predicted			
I	1061	937	1000	980	960
II	130	123	100	139	ND
III	594	548	580	510	458
IV	281	208	200	240	189
V	658	622	580	640	543
V2	1316	1244	1130	1280	1231

* Castañeda-Tamez 2024 [5], ** Matuz-Mares 2021 [10], *** Guerrero- Castillo 2009 [39], ND, Not determined.

## Data Availability

The original contributions presented in the study are included in the article/supplementary material, further inquiries can be directed to the corresponding author/s.

## Supplementary Materials

The following supporting information can be downloaded at: https://www.mdpi.com/article/10.3390/microorganisms12101931/s1, Supplementary S1: Bioinformatic analysis. Supplementary S2: Abundance of monomer and dimer of the ATP synthase.
